# The Need to Consider Geochemistry When Interpreting Sr-Isotopes. Comment on Gregorčič et al. The Provenance of Slovenian Milk Using ^87^Sr/^86^Sr Isotope Ratios. *Foods* 2021, *10*, 1729

**DOI:** 10.3390/foods11040564

**Published:** 2022-02-16

**Authors:** Micha Horacek

**Affiliations:** 1 Department of Lithospheric Research, Vienna University, 1090 Vienna, Austria; untertrias@gmail.com; 2 HBLFA Francisco-Josephinum-BLT Wieselburg, 3250 Wieselburg, Austria

I was very interested in the investigation of the ^87^Sr/^86^Sr ratio of Slovenian milk by Gregorcic et al. (2021) [1]. As Slovenia is a very geologically diverse country, the differentiation of geographic origin by strontium isotopes would be a very important result, as it should be transferable to different food commodities, as this proxy usually isn’t significantly influenced by plant or animal metabolisms. However, classification of the milk samples, based on the geological age of the bedrock, was done very impractically and in an unfortunate way. Also, some interpretations do not seem probable.

Generally, the geological age of the bedrock does not necessarily indicate a certain ^87^Sr/^86^Sr ratio, and furthermore, the ^87^Sr/^86^Sr ratio is far more influenced by the type of bedrock (the kind of rock, e.g., carbonate, clay, basalt, granite, etc.) than the age of bedrock.

## Sample Grouping

Based on the geological situation of Slovenia, as shown by Gregorcic et al. (2021) [1], a far more logical grouping of the samples would be as follows: samples from north-eastern Slovenia (including Paleogene, Neogene, and Quaternary samples, all dominated by clastic (siliciclastic) deposits, according to [1]), and carbonate-dominated areas of all other regions. Taking into account additional isotopic parameters (e.g., H, O, C), a further subdivision of the carbonate areas into central (alpine?) Slovenia (including the Triassic and Jurassic samples) and coastal Slovenia (Cretaceous samples) seems reasonable, to evaluate potential climatically and topographically induced differences. It seems that later on, such a grouping was done anyway for statistical evaluation, but to me, it would be much more logical (because defined by regions) to have such a grouping from the start. Consequently, statistical evaluation of the differentiation of the initial grouping solely according to ^87^Sr/^86^Sr ratio does not seem to have been successful, as this isn’t even shown. Such an evaluation was done only in combination with further isotopic parameters analyzed.

## ^87^Sr/^86^Sr in Milk

**Figure 1:**
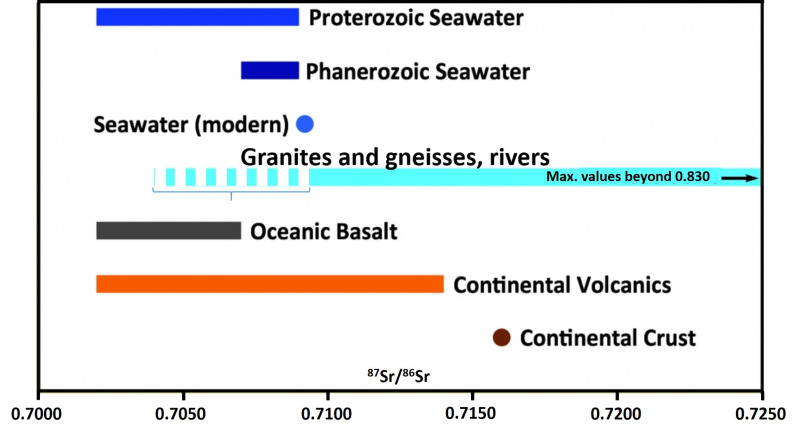
^87^Sr/^86^Sr ratios of different rock and water types. Seawater ^87^Sr/^86^Sr ratios are the result of interplay of Sr transfer from oceanic basalts and transport of Sr from the continents (erosion of granites and gneisses) via rivers into the sea. Marine carbonates incorporate the ^87^Sr/^86^Sr ratio of the ambient seawater and thus have the same ratio. Point “Continental Crust” indicates average value. Bracket marks the isotope interval where rivers and gneisses ^87^Sr/^86^Sr ratios are, if dominantly influenced by oceanic basalts or marine carbonates. Data accumulated after [2,3,4].

**Figure 2:**
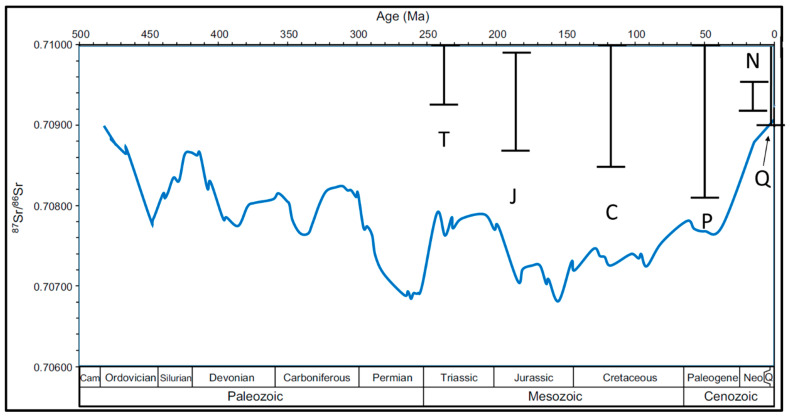
Seawater ^87^Sr/^86^Sr curve modified after [2], with the curve giving 1 Ma averaged increments. Whiskers show the milk isotope data by Gregorcic et al. (2021) [1]. T: Triassic, J: Jurassic, C: Cretaceous, P: Paleogene, N: Neogene, Q: Quaternary. Missing upper whiskers indicate milk ^87^Sr/^86^Sr ratios beyond the scale.

As already stated, instead of geological age, the type of bedrock (carbonate, siliciclastic, metamorphic, and magmatic rock) is the dominant influence on the ^87^Sr/^86^Sr ratio of soil (Figure 1). Marine (Phanerozoic) carbonates show ^87^Sr/^86^Sr ratios within a very well-defined range (within 0.7068–0.7092 [2], Figure 2). Siliciclastic rocks usually possess notably higher ^87^Sr/^86^Sr ratios with respect to carbonates, whereas igneous/magmatic/metamorphic rocks can be either depleted (basaltic rocks) or also enriched (acidic-magmatic and metamorphic rocks) [2,3,4]. Still, although marine carbonates are that restricted in ^87^Sr/^86^Sr ratio variations, often the soil in carbonate bedrock areas possesses higher ^87^Sr/^86^Sr ratios than the marine carbonate bedrock range. This is because during erosion and soil formation, carbonate is often/usually removed by chemical erosion (dissolution), and the “eroded” material is then removed by the dissolving water. In this way, siliciclastic impurities and intervals in the carbonate succession, and aeolian sediments (e.g., loess) are enriched in the soil covering the carbonate bedrock; thus, the soil (and consequently the plants as well as animals feeding on these plants) can possess higher ^87^Sr/^86^Sr ratios than the bedrock. This is also the case in Gregorcic et al. (2021) [1] (Figure 2). Gregorcic et al. (2021) [1] explain that elevated ^87^Sr/^86^Sr ratios in milk with respect to ambient river water (their Figure 5) could be due to the potential addition of lime for soil improvement. However, this explanation is invalid in the present case, as the referenced article [5] documents a lowering of the ^87^Sr/^86^Sr ratio due to lime (most probably marine (calcite) carbonate) addition to low-/non-calcareous soils. The data of Gregorcic et al. (2021) [1], however, document an increase in the ^87^Sr/^86^Sr ratio of most probably calcareous soils (evidenced by the ^87^Sr/^86^Sr ratios; an identification of the data in the Figure is not possible). Thus, as stated above, an increase in the ^87^Sr/^86^Sr ratio of the milk with respect to the ambient bedrock geology and river water being due to the influence of siliciclastic material/sediments is the more probable explanation. This result supports the interpretation that the ^87^Sr/^86^Sr ratio of milk is dominantly influenced by the feed instead of the water (as the latter (in carbonate bedrock) is usually dominated by the carbonate bedrock ^87^Sr/^86^Sr ratio due to the dissolved carbonate).

**Figure 3:**
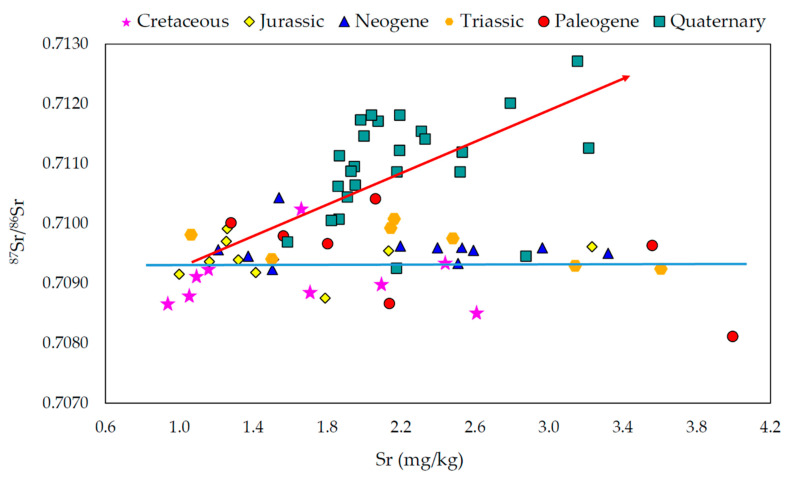
^87^Sr/^86^Sr ratios of milk reported by Gregorcic et al. (2021) [1] versus Sr concentrations. Blue line approximates Trend 1: increasing Sr concentrations with constant ^87^Sr/^86^Sr ratio. Red arrow indicates Trend 2: increasing Sr concentrations with increasing ^87^Sr/^86^Sr ratios.

To explain the two trends identified in the milk ^87^Sr/^86^Sr ratio and Sr concentration data, Gregorcic et al. (2021) [1], (Figure 3) outline two processes: “(i) different weathering rates of specific minerals in the rocks and soils; movement of water and sediments in a grazing area can influence Sr and Rb content of milk samples, potentially leading to different ^87^Sr/^86^Sr isotope ratios; (ii) the consumption of imported plants, particularly those enriched with high Ca and Sr content, …”. However, the most plausible explanation for these trends is in fact as follows: Trend 1 (no change in ^87^Sr/^86^Sr ratio with increasing Sr concentrations, Figure 3) indicates different amounts of marine Sr in the carbonate bedrock, which can most likely be explained by carbonate mineralogy and chemistry. Synchronously formed marine carbonates can possess highly variable Sr concentrations, but with the same ^87^Sr/^86^Sr ratio (e.g., [6] supplementary materials). Trend 2 (increase in ^87^Sr/^86^Sr ratio with increasing Sr concentrations, Figure 3) documents a mixing line from (+/− marine carbonate) low ^87^Sr/^86^Sr ratio towards high ^87^Sr/^86^Sr ratio, with increasing amounts/influence of siliciclastics, the latter in the present case most likely coming from areas with Precambrian and Palaeozoic metamorphic and igneous bedrock (north-eastern Slovenia). Of course, imported feed, or feed coming from a locality with significantly different geology, can potentially play a significant role in changing the ^87^Sr/^86^Sr ratio of milk (e.g., with respect to summer/winter), as imported feed can potentially overprint the local ^87^Sr/^86^Sr ratio (and modify the Sr concentration). However, in the data presented by Gregorcic et al. (2021) [1], differing ^87^Sr/^86^Sr ratios between summer and winter values could be documented for only a few of the farms sampled (e.g., C5, Q4). Thus, this explanation by Gregorcic et al. (2021) [1] cannot satisfactorily explain the two identified trends.

Application of ^87^Sr/^86^Sr ratio for determination and control of geographic origin can be a very potent tool, depending on the exact question intended to be answered. Areas with homogenous bedrock geology and variations in ^87^Sr/^86^Sr ratio are very well suited, whereas heterogeneous bedrock geological settings are a challenge for this method, as outliers and heterogeneous values within individual areas are to be expected. One must keep in mind that the highest ^87^Sr/^86^Sr ratio for Slovenian truffles [1,7] was reported from central Slovenia dominated by Triassic and Jurassic carbonate bedrock.

## Data Availability

Data is contained within the article.

## References

[1] Gregorčič S.H., Ogrinc N., Frew R., Nečemer M., Strojnik L., Zuliani T. (2021). The Provenance of Slovenian Milk Using ^87^Sr/^86^Sr Isotope Ratios. Foods.

[2] Zaky A.H., Brand U.U., Buhl D., Blamey N., Bitner M.A., Logan A., Gaspard D., Popov A., Bitner A. (2019). Strontium isotope geochemistry of modern and ancient archives: Tracer of secular change in ocean chemistry. Can. J. Earth Sci..

[3] Allegre C.J. (2008). Isotope Geology.

[4] White W.M. (2015). Isotope Geochemistry.

[5] Thomsen E., Andreasen R. (2019). Agricultural lime disturbs natural strontium isotope variations: Implications for provenance and migration studies. Sci. Adv..

[6] Sedlacek A.R., Saltzman M.R., Algeo T.J., Horacek M., Brandner R., Foland K., Denniston R.F. (2014). ^87^Sr/^86^Sr stratigraphy from the early triassic of Zal, Iran: Linking temperature to weathering rates and the tempo of ecosystem recovery. Geology.

[7] Gregorčič S.H., Strojnik L., Potoˇcnik D., Vogel-Mikuš K., Jagodic M., Camin F., Zuliani T., Ogrinc N. (2020). Can we discover truffle’s true identity?. Molecules.

